# Cell Type-Specific Dependency on the PI3K/Akt Signaling Pathway for the Endogenous Epo and VEGF Induction by Baicalein in Neurons versus Astrocytes

**DOI:** 10.1371/journal.pone.0069019

**Published:** 2013-07-19

**Authors:** Yu-Yo Sun, Shang-Hsuan Lin, Hung-Cheng Lin, Chia-Chi Hung, Chen-Yu Wang, Yen-Chu Lin, Kuo-Sheng Hung, Cheng-Chang Lien, Chia-Yi Kuan, Yi-Hsuan Lee

**Affiliations:** 1 Division of Neurology, Department of Pediatrics, the Center for Neurodegenerative Disease, Emory University School of Medicine, Atlanta, Georgia, United States of America; 2 Department and Institute of Physiology, National Yang-Ming University, Taipei, Taiwan; 3 Graduate Institute of Medical Sciences, Taipei Medical University, Taipei, Taiwan; 4 Brain Research Center, National Yang-Ming University, Taipei, Taiwan; 5 Institute of Neuroscience, National Yang-Ming University, Taipei, Taiwan; 6 Department of Neurosurgery, Taipei Medical University Wan Fang Hospital, Taipei, Taiwan; CNRS UMR7275, France

## Abstract

The neuroprotective effect of baicalein is generally attributed to inhibition of 12/15-lipoxygenase (12/15-LOX) and suppression of oxidative stress, but recent studies showed that baicalein also activates hypoxia-inducible factor-α (HIF1α) through inhibition of prolyl hydrolase 2 (PHD2) and activation of the phosphatidylinositide-3 kinase (PI3K)/Akt signaling pathway. Yet, the significance and regulation of prosurvival cytokines erythropoietin (Epo) and vascular endothelial growth factor (VEGF), two transcriptional targets of HIF1α, in baicalein-mediated neuroprotection in neurons and astrocytes remains unknown. Here we investigated the causal relationship between the PI3K/Akt signaling pathway and Epo/VEGF expression in baicalein-mediated neuroprotection in primary rat cortical neurons and astrocytes. Our results show that baicalein induced Epo and VEGF expression in a HIF1α- and PI3K/Akt-dependent manner in neurons. Baicalein also protected neurons against excitotoxicity in a PI3K- and Epo/VEGF-dependent manner without affecting neuronal excitability. In contrast, at least a 10-fold higher concentration of baicalein was needed to induce Epo/VEGF production and PI3K/Akt activity in astrocytes for protection of neurons. Moreover, only baicalein-induced astrocytic VEGF, but not Epo expression requires HIF1α, while PI3K/Akt signaling had little role in baicalein-induced astrocytic Epo/VEGF expression. These results suggest distinct mechanisms of baicalein-mediated Epo/VEGF production in neurons and astrocytes for neuroprotection, and provide new insights into the mechanisms and potential of baicalein in treating brain injury *in vivo*.

## Introduction

Baicalein, a natural flavonoid isolated from *Scutellaria* *baicalensis* Georgi (S. Georgi), has been shown effective in attenuating neuronal loss induced by excitotoxin administration [1] and oxygen-glucose deprivation [2] *in vitro* as well as reducing brain injury in various brain injury animal models [2–4]. The mechanism of action for the baicalein neuroprotection has been mostly attributed to its direct inhibition of 12/15 lipoxygenase (12/15-LOX), which is mainly expressed in neurons and brain cerebrovascular endothelial cells and is involved in injury-induced elevation of reactive oxygen species and subsequent lipid peroxidation causing neural cell necrosis (reviewed by [5–7]) and blood-brain barrier (BBB) disruption [8]. Recent studies revealed that baicalein also regulates other signaling pathways, including prolyl hydroxylase 2 (PHD2)/hypoxia-inducible factor 1α (HIF1α) [9] and phosphatidylinositide 3-kinase (PI3K)/Akt pathways [2]. However, how these pathways integrate to provide neuroprotection remains poorly understood.

PI3K/Akt signaling was reported to be activated by baicalein in neurons, and plays a key role in baicalein-mediated neuronal survival and synaptic plasticity [2,10]. Akt is mainly phosphorylated by class I PI3Ks and plays important roles in neuronal survival [11,12]. The PI3K/Akt signaling pathway activates HIF1α by reducing its ubiquitination via two routes, one by phosphorylation of HIFα, and the other by inhibition of PHD2 via the mammalian target of rapamycin (mTOR) [13,14]. PHD2, one of the 3 PHD isoforms (PHD1, PHD2, PHD3) that serve as intracellular oxygen sensors, mediates asparaginyl hydroxylation and ubiquitination of HIF-1α upon normoxic condition [15,16]. Thus, compounds or signaling pathways that inhibit PHD activity can also up-regulate HIF-1α under normoxia. Recent studies show that both neuron-specific PHD2 knockout and PHD2 inhibitor treatment are effective in reducing transient cerebral ischemia-induced brain damage via activating HIF-1α [17,18]. Notably, baicalein can inhibit PHD2 activity by direct binding to the enzyme active sites [9], but whether its activation of prosurvival PI3K/Akt signaling in neurons also contributes to the HIF1α target gene induction remains undetermined.

Erythropoietin (Epo) and vascular endothelial growth factor (VEGF) are hypoxia-inducible neuroprotective cytokines with their gene transcription mainly mediated by HIF-1α or HIF-2α [15]. Recent efforts in the development of neuroprotective therapeutics have been directed to the induction of endogenous Epo and VEGF using HIF-activating agents, such as ischemic preconditioning [19] and PHD2 inhibitors [20], for treating CNS injury in order to circumvent the possible adverse effects of their exogenous application [21–25]. While the induction of endogenous Epo from brain cells was reportedly beneficial [26,27], controversial outcomes were noted regarding the endogenous VEGF induction: neuronal VEGF production appears to be neuroprotective [28,29] whereas excessive astrocytic VEGF was found detrimental to the BBB integrity [30]. Baicalein, as a PHD2 inhibitor and neuronal PI3K activator, seem to be a promising candidate for inducing Epo/VEGF in the brain, but such an effect and a subsequent contribution to baicalein neuroprotection have not been investigated.

Most of studies on baicalein neuroprotection were focusing on its neuronal effects, such as 12/15-LOX inhibition, PI3K activation, and regulation of GABA_A_ receptor activity [31], whereas its effect on astrocytes, the most abundant cell type in the brain, has not been well investigated. Factors released from astrocytes, including neurotrophic factors and proinflammatory cytokines, vary under different physiological and pathological settings and play important roles in establishing a microenvironment that affects neuronal survival and plasticity [32]. The PI3K/Akt signaling pathway in astrocytes was found to be important for the glutamate transporter function [33] and the synthesis of an astrocyte-derived neuroprotective chemokine RANTES [34], but its effects on other neuroprotective factors have not been explored. Besides, the effect of baicalein on PI3K activity in astrocytes has not been reported to date while it is quite variable across different cell types: it is stimulatory in neurons but inhibitory in microglia and prostate cancer cells [35,36].

In this study, we investigated the causal role of baicalein-induced PI3K/Akt signaling in its activation of HIF1α and downstream Epo/VEGF gene expression in primary cortical neurons and astrocytes. Our data show that baicalein activates PI3K/Akt signaling in both neurons and astrocytes but at different effective concentrations to provide neuroprotection against excitotoxicity, and notably this signaling only contributes to its induction of neuronal, but not astrocytic, Epo/VEGF expression. Furthermore, the VEGF-inducing effect of baicalein requires its activation of HIF1α in both cell types, whereas its Epo-inducing effect only depends on HIF1α in neurons, but not (in) astrocytes. The contributions of 12/15-LOX and PHD2 in the baicalein-induced neuronal and astrocytic Epo/VEGF expression were also examined. Clues for the potential application of baicalein in treating brain injuries and diseases based on the information obtained are discussed. 

## Materials and Methods

### Reagents

Baicalein was obtained from Merck (Darmstadt, Germany). L-glutamic acid, N-methyl-D-aspartic acid (NMDA), bicucullin, and cobalt chloride (CoCl_2_) were obtained from Sigma-Aldrich (St. Louis, MO). LY294002 was obtained from Calbiochem (San Diego, CA). PI3K α inhibitor 2 (3-[4-(4-morpholinyl)thieno[3,2-d]pyrimidin-2-yl-phenol) and the PI3Kγ inhibitor CAY10505 (5-[[5-(4-fluorophenyl)-2-furanyl] methylene]-2,4-thiazolidinedione) were obtained from Cayman Chemical (Ann Arbor, MI). Dimethyloxaloylglycine (DMOG), a PHD2 inhibitor, was obtained from Enzo Life Sciences (Plymouth Meeting, PA). Goat IgG, goat anti-VEGF and goat anti-Epo antibodies for neutralization study were purchased from R&D Systems (Minneapolis, MN).

### Animals

For the primary culture of cortical neurons and astrocytes, we used pregnant female Sprague Dawley (SD) rats at 17-day gestation and postnatal 1-2-day old (P1-P2) SD rats obtained from BioLASCO Taiwan Co. (Taipei, Taiwan), respectively. For the rat hippocampal slice preparation, SD male rats at P16-P21 were used. Animals used for primary cultures and for hippocampal slice preparation were killed by overdose sevoflurane (Abbott, Osaka, Japan). Animal experimentation procedures were reviewed and approved by the Animal Care and Use Committee at National Yang-Ming University and are in accordance with the Guide for the Care and Use of Laboratory Animals, the National Institute of Health guidelines (USA) in the care and use of animals for experimental procedures.

### Primary cultured rat cortical neurons and astrocytes

The cultured cortical neurons were prepared from fetal rats harvested from pregnant female rat at 17-day gestation as described previously [37]. Briefly, rat brain cortics was loosely homogenized through a 14-gauge metal needle in BME (Invitrogen) with sodium bicarbonate (26.2 mM), D-glucose (27.8 mM), L-glutamine (2.0 mM), and 20% FBS (Invitrogen), centrifuged at 800 rpm for 5 min, and washed three times. Resuspended cells were seeded onto cell culture plates (35-mm culture dish or 24-well culture plates; Iwaki, Tokyo, Japan) pre-coated with poly-L-lysine (Sigma-Aldrich, St. Louis, MO), and then incubated in 37°C incubator with 5% CO_2_ for 30~45 min, after which the medium was replaced by Neural Basal medium (Invitrogen) with sodium bicarbonate (26.2 mM), D-glucose (27.8 mM), and L-glutamine (2.0 mM). The obtained neuron-enriched cultures at 10 days–*in-vitro* (DIV) contained more than 85% neuronal population as characterized by immunofluorescent double labeling of neuron and glial markers (Figure S1 in Information S1). Cultured neurons at this stage were sensitive to NMDA excitotoxicity [37], and thus used in this study.

Primary cultured astrocytes were prepared from P1-P2 SD rats as described previously [38]. Briefly, cerebral cortex isolated from neonatal rats was loosely homogenized through a 14-gauge metal needle in DMEM/F12 (Invitrogen) with 10% FBS, filtered through a 70-µm nylon mesh, and centrifuged at 1,000 rpm for 10 min. Cells resuspended were seeded onto 75 mm flasks and incubated for 7 days, followed by orbital shaking at 180 rpm in a 37°C incubator for 24 h to remove microglia and oligodendrocytes. The purified astrocytes that tightly adhered at the bottom of the flasks were then detached with trypsin/EDTA (Invitrogen) and seeded onto culture dishes and incubated for 7 days to settle to a resting stage. The purified astrocyte cultures which contained more than 85% glial fibrillary acidic protein (GFAP)-positive cells were used for this study.

### Plasmid construction and luciferase activity assay

A DNA fragment with triplicated hypoxia response element (HRE) sequences in the human *EPO* gene enhancer (HRE_*EPO*_) was inserted into the promoter of pGL2 luciferase reporter plasmid (Promega, Madison, WI) to obtain the pHRE_*EPO*_-Luc reporter construct as described previously [27]. Cells plated in 24-well plates were transfected with pHRE_*EPO*_-Luc at 0.33 μg/well and pRL-TK *Renilla* luciferase normalization construct (Promega) at 0.01 μg/well in 1.5 μg/ml Lipofectamine 2000^TM^ (Invitrogen, Carlsbad, CA) for 24 h, followed by treatment with baicalein or CoCl_2_ for 24 h. Cells were then harvested with passive lysis buffer (Promega) for the luciferase activity assay according to the manufacture’s protocol (Dual-Luciferase^®^ reporter assay system; Promega). The HRE_*EPO*_-driven gene expression was calculated and represented as the ratio of firefly/renilla luciferase activity. No significant cytotoxicity was found with the concentration of Lipofectamine 2000^TM^ used in this study.

### Semi-quantitative and real-time RT-PCR

Total RNA was extracted using TRIzol reagent (Invitrogen), and reversely transcribed by using High Capacity cDNA Reverse Transcription Kit (Applied BioSystems, Foster City, CA) to obtain cDNAs for subsequent semi-quantitative and quantitative PCR, The semi-quantitative PCR of rat *Epo, Vegf*, *Hif1a* (HIF-1α), *Alox15* (12/15-Lox), and a housekeeping gene *Gapdh* (glyceraldehydes-3-phosphate dehydrogenase) cDNAs were detected using the following primers: *Epo*, 5'-TGCGACAGTCGCGTTCTGGAGAGGTAC-3’and5'-ATCCGCTGTGAGTGTTCGGAGTGGAGC-3′; *Vegf*, 5'-CCATGAACTTTCTGCTCTCTTG-3’ and 5'-GGTGAGAGGTCTAGTTCCCGA -3′; Hif1a, 5'-CAAGATCAGCCAGCAAGTCCTTCTGATG-3’ and 5'-AGGTTTCTGTAACTGGGTCTGCTGGAATC-3′; *Alox15*, 5'-GACTGTTCAGGAAACATAGGGAAG-3′ and 5'-CCATTACCCCTATAACCTGTGAAG-3′; *Gapdh*, 5'-CTCATGACCACAGTCCATGC-3′ and 5'-TTCAGCTCTGGGATGACCTT-3′. Bands of PCR products were visualized and quantified using an electrophoresis image analysis system (Eastman Kodak Co., Rochester, NY). For the quantitative real-time PCR, the FAM probes and primers for the detection of *Epo* (Rn00566529_m1; 5’-GAGATGGGGGTGCCCGAACGTCCCA-3’) and an internal control gene *β-actin* (Rn00667869_m1; 5’-CTTCCTGGGTATGGAATCCTGTGGC-3’) were designed by Applied Biosystems (ABI, Foster City, CA) and used for TaqMan system. Primer sets for *Vegf* (5’-CGGACGGGCCTCTGAAACCAT-3’ and 5’-CTTCACCACTTCATGGGCTTTCTGC-3’), TNFα (5’-TCTCAAAACTCGAGTGACAAGCCCG-3’ and 5’-GCAGCCTTGTCCCTTGAAGAGAACC-3’), and internal control *Gapdh* (5'-CTCATGACCACAGTCCATGC-3′ and 5'-TTCAGCTCTGGGATGACCTT-3′) were used for SYBR Green system. The assay mixture and 150 ng of cDNA were added into 2x TaqMan^®^ Universal PCR Mix or 2x SYBR^®^ Green PCR Master Mix (Applied Biosystems) to make up 20 μl of the amplification mixtures, and then subjected to real-time PCR reaction on an ABI PRISM 7300 Sequence Detector. The average cycle threshold (Ct) value was used to calculate mRNA expression levels. Relative mRNA levels were normalized by the internal control (*ß-actin or Gapdh*) in terms of the differences of the C_t_ values. Relative transcript levels were calculated as x = 2^-△Ct^, in which ΔCt = Ct_target gene_ –Ct_internal control_.

### Enzyme-linked immunosorbent assay (ELISA)

In brief, culture media of cortical neurons and astrocytes were collected after the treatment, and the cells were lysed in a lysis buffer [20 mM Tris, pH 7.4, 150 mM NaCl, 1% IPGEAL-630, 5% glycerol, protease inhibitor cocktail (Roche, Penzberg, Germany)]. Epo and VEGF concentrations in the cell lysate and culture media were measured using the respective Quantikine ELISA Kits (R&D Systems, Minneapolis, MN) according to the manufacturer’s instructions, and detected using an ELISA reader at a wavelength of 450 nm.

### RNA knockdown

Cortical neurons were transfected with siRNAs specific for rat 12/15-LOX or HIF-1α mRNA, or with scrambled RNA produced by Silencer Pre-designed siRNA (Ambion, Austin, TX) using Lipofectamine 2000^TM^ reagent (Invitrogen) for 72 h as previously described [39]. The siRNA sequences for rat 12/15-LOX (*Alox15*; Accession No. NM_031010) were 5’-CGAUUUCGAGAGGACAAAAtt-3’ (exon 4) and 5’-GGCAGAUCAUGAAUCGGUAtt-3’ (exon 10); for rat HIF-1α (*Hif1a*; Accession No. NM_024359) were 5’-gcuugcucaucaguugccatt-3’ (exon 2) and 5’-CCAGUUGAAUCUUCAGAUtt-3’ (exon 9). The knockdown efficiency of each gene product was examined by semi-quantitative RT-PCR.

### Western blot analysis

Cortical neurons were lysed and total protein extracted for Western blot analysis as described previously [39]. Primary antibodies used were rabbit anti-phospho-Akt (Ser473) (1:1000) and rabbit anti-Akt (1:1,000) antibodies (Cell Signaling, Danvers, MA), and secondary antibodies were horseradish peroxidase (HRP)-conjugated goat anti- rabbit IgG (1:20,000) and HRP-conjugated goat anti- mouse IgG (1:20,000) (Jackson ImmunoResearch Laboratories, West Grove, PA). The immune complex was visualized by HRP-reactive Western Lightning^TM^ *Plus*-ECL (PerkinElmer Inc., Waltham, MA) and the signal was detected and analyzed by Night OWL LB 981 imaging system (Berthold Technologies, Bad Wilbad, Germany).

### Chromatin immunoprecipitation (ChIP) assay

Anti-HIF1α-based ChIP assay was performed as described previously [27]. DNA-protein cross-linking samples were subjected to immunoprecipitation using mouse anti-HIF1α antibody (2 μg; Novus, Littleton, CO). Purified DNA was amplified by PCR with primer for HRE-specific rat *Epo* gene 3’ enhancer (+3497 ~ +3618, NM-017001, 5’-TACCTCCCCCCCCCCCCATTCTGGT-3’ and 5’-CAAGCCCAGAGGGGTCAAGAGGTCAGA-3’), rat *Epo* gene promoter (-375 ~ -221, NM-017001, 5’-CAGCCTGCTCTACCCCAGCAAGGA-3’ and 5’-GGGGGTCGGGGATGTTATCAGCA-3’), and HRE-specific rat *Vegf* gene promoter (-1829 ~ -1994, M-32167, 5’-GAGGAACAAGGGCTTCTGTCTG-3’ and 5’-TCTCTGGAGAGGATATGGCATC-3’). Quantitative real-time PCR of each gene promoter and enhancer fragment was performed using SYBER Green reaction mix (Applied Biosystems).

### Cell apoptosis analysis

Cell apoptosis analysis was performed using 4′,6-diamidino-2-phenylindole dihydrochloride (DAPI) staining for DNA condensation and terminal deoxynucleotidyl transferase dUTP nick end labeling (TUNEL) for fragmented DNA. Cells were briefly washed with isotonic saline solution, followed by fixation (4% formaldehyde in 20 mM PBS) for 15 min at room temperature and permeabilization with pre-chilled EtOH/CH3COOH (95%: 5%) for 15 min at –20^o^C. Cells were then incubated with nuclear marker DAPI. TUNEL stain was performed following the manufacturer’s protocol (Promega). Fluorescent micrographs at excitation wavelength 350 nm for DAPI and 488 nm for TUNEL were taken by Olympus DP50 digital camera (Olympus, Tokyo, Japan). Apoptotic cells were identified by visualizing TUNEL-positive cells with condensed DAPI staining in the nucleus. The total number of DAPI-stained cells served as the total cell number. The cell counting was performed in five randomly selected areas for each well and 4 separate wells were used for each experimental condition. The number of apoptotic cells in each well was divided by its respective total cell number to obtain the percent of cell apoptosis.

### Electrophysiology

Transverse hippocampal slices (300 μm) were prepared from male SD rats at P16-P21 using a Microslicer (DTK -1000, Dosaka, Kyoto, Japan). Slices were sectioned in the ice-cold cutting buffer containing (in mM): 87 NaCl, 25 NaHCO_3_, 1.25 NaH_2_PO_4_, 2.5 KCl, 10 glucose, 75 sucrose, 0.5 CaCl_2_ and 7 MgCl_2_. The slices were recovered (25 min, 34 ˚C) in the cutting buffer oxygenated with 95% O_2_/5% CO_2_, and then stored at room temperature. During experiments, each slice was transferred to a submersion recording chamber and was superfused with oxygenated artificial cerebrospinal fluid (ACSF) containing (in mM): 125 NaCl, 25 NaHCO_3_, 1.25 NaH_2_PO_4_, 2.5 KCl, 25 glucose, 2 CaCl_2_, and 1 MgCl_2_.

Patch pipettes were pulled from borosilicate glass tubing (outer diameter 1.5 mm, inner diameter 0.86 mm; Harvard apparatus, Holliston, MA) and heat-polished before used. Both CA1 pyramidal cells and dentate granule cells were visually selected for whole-cell patch recordings (pipette resistance 3-5 MΩ) under differential interference contrast optics (BX51WI, Olympus, Tokyo, Japan) using Multiclamp 700B or Axopatch 200B amplifiers (Molecular Devices, Union City, CA) as described [40]. Pipette capacitance was carefully compensated to >95%. Series resistance (about 12-17 MΩ) was compensated to >95% in current-clamp configuration and >80% in voltage-clamp configuration. Stability of series resistance was continuously monitored throughout the experiments. Signals were low-pass filtered at 5 kHz (four-pole Bessel), and sampled at 10 kHz using the Digidata 1440 (Molecular Devices); data acquisition and pulse generation were performed using pClamp 10.2 (Molecular Devices). Recordings were made at 22-24 ˚C.

For miniature recordings, recording pipettes were filled with Cl^-^-rich internal solution, containing (mM): 25 K-gluconate, 140 KCl, 0.3 EGTA, 4 MgATP, 10 Hepes, 10Na_2_-phosphocreatine; pH adjusted to 7.3 with KOH; otherwise, the internal solution contained (mM): 135 K-gluconate, 20 KCl, 0.1 EGTA, 2 MgCl_2_, 4 Na _2_ATP, 10 Hepes and 0.3 Na _3_GTP; pH adjusted to 7.3 with KOH. Kynurenic acid was obtained from Sigma; tetrodotoxin (0.5 μM) from Tocris Bioscience (Bristol, UK) was added in the miniature current recordings. All other chemicals were purchased from Sigma (St. Louis, MO) except where noted.

### Preparation of astrocyte-conditioned medium (ACM)

Astrocyte-conditioned medium (ACM) was collected from astrocyte cultures treated with baicalein or LY294002 plus baicalein for 24 h, and then filtered through Amicon® Ultra-0.5 Centrifugal Filter Devices (Millipore, Billerica, MA) with 10-kDa molecular weight cut-off to remove the small molecule compounds and concentrate the astrocyte-derived protein factors (>10 kDa) in the retentate. The ACM retentate was then reconstituted to the original volume with fresh culture medium, and then the above dialysis procedure was repeated twice. The final round of ACM retentate reconstituted to the original volume contained 1000x diluted small molecule compounds while preserving the astrocyte-derived protein cytokines/factors at their original concentration, and was used for treating cortical neurons.

### Statistics

Statistical analysis was performed using GraphPad Prism® 5 software (GraphPad Software, San Diego, CA). For electrophysiology experiments, data were analyzed using Clampfit 10.2 (Molecular Devices) and GraphPad Prism 5.0. The input resistance was determined from the voltage at the end of the 1-s hyperpolarizing current pulse (-100 pA). Data are expressed as mean ± SEM. Statistical analysis was performed by one-way ANOVA to evaluate the difference among all groups, followed by Dunnet’s, Newman-Keuls multiple-comparisons post hoc test to compare designated pairs of groups. For electrophysiology data, statistical analysis was assessed using a two-sided Wilcoxon signed rank test for paired samples. Statistical significance was assumed at *p* < 0.05.

## Results

### Baicalein activates HIF1α to increase Epo and VEGF expression in cortical neurons

**Figure 1:**
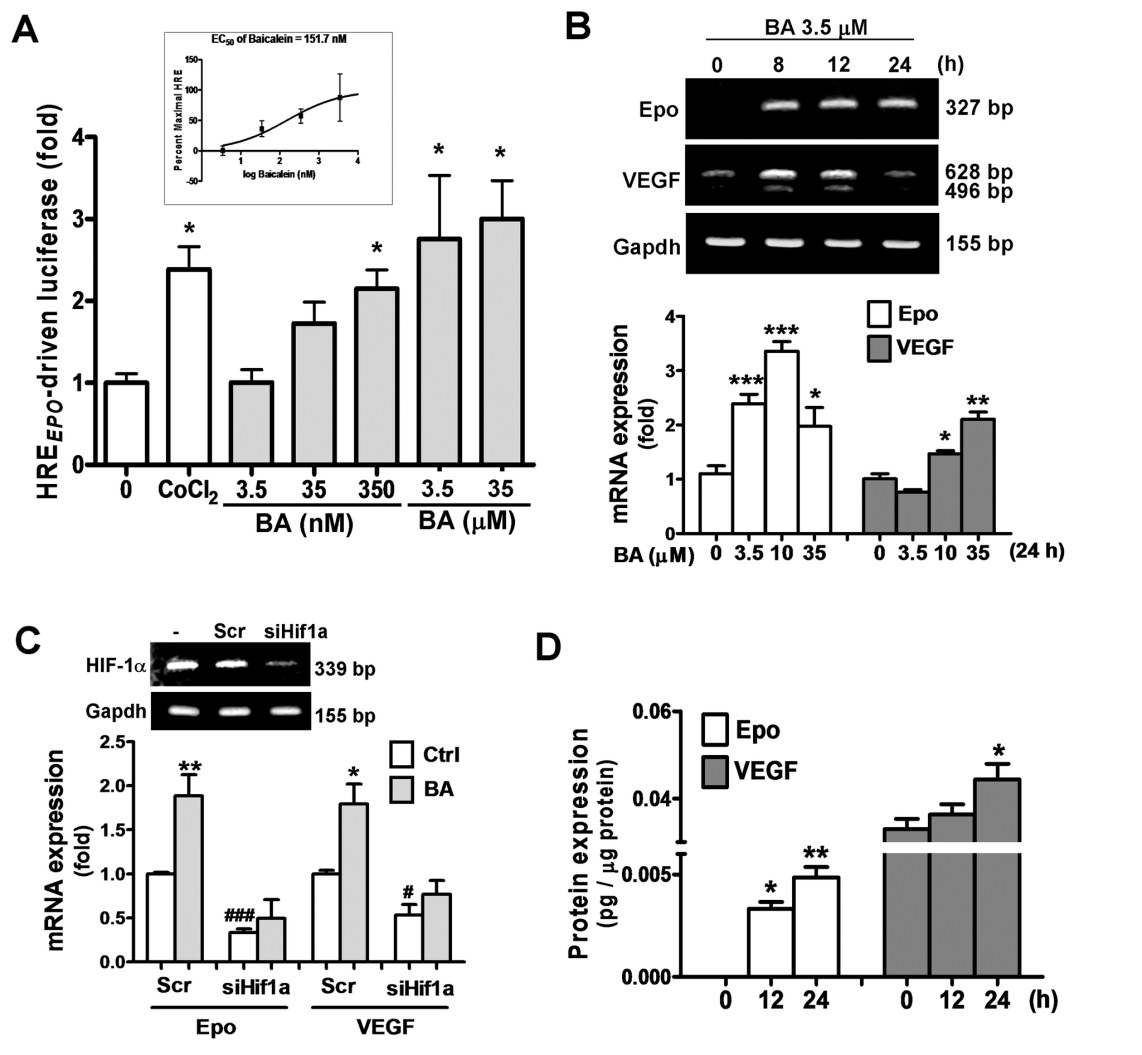
Effects of baicalein on the HIF1α activity and expression of Epo and VEGF in cortical neurons. (A) Primary cultured cortical neurons co-transfected with pHRE_*EPO*_-Luc and pRL-TK were treated with baicalein (BA) at indicated concentrations (3.5 nM~35 μM) or CoCl_2_ (0.4 mM) for luciferase activity assay of HRE-driven gene expression as an index of HIF activity. Inset in A: EC50 of BA on HRE_*EPO*_-driven luciferase. (B) qRT-PCR of Epo and VEGF mRNA of RNA extracted BA-treated neurons at indicated time or concentrations. (C) Upper panel: RT-PCR analysis of HIF1α mRNA in neurons transfected with scrambled RNA (Scr) or siHif1a. Lower panel: qRT-PCR analysis of Epo and VEGF mRNA in 3.5 μM BA-treated neurons transfected with Scr or siHif1a. (D) ELISA of Epo and VEGF of cell lysate of BA-treated neurons. Data represent means ± SEM (n=3). *p<0.05, **p<0.01 and ***p<0.001 versus vehicle-treated control by one-way ANOVA and Newman-Keuls multiple comparison posttest; ^#^ *p*<0.05 and ^# # #^ *p*<0.001 versus the Scr-Ctrl by unpaired *t*-test.

We first examined whether baicalein activates HIFs in primary cultured cortical neurons by using a luciferase reporter construct with a triplicate HRE DNA fragment from the human *Epo* gene enhancer (pHRE_*EPO*_-Luc). Figure 1A shows that baicalein treatment dose-dependently increased pHRE_*EPO*_-Luc activity, ranging from 3.5 nM to 35 μM, with a minimal effective concentration at 35 nM, minimal concentration for maximal response at 3.5 μM, and an EC 50 of 151.7 nM (Figure 1A inset). The baicalein-increased HRE_*EPO*_-Luc activity was comparable to the activity induced by the hypoxia mimetic cobalt chloride (CoCl_2_). Time course results show that baicalein caused an increase in Epo mRNA at 8 h that was sustained for up to 24 h; whereas VEGF mRNA was increased transiently (Figure 1B upper panel). The dose dependent effect of baicalein on the Epo/VEGF expression was also similar to its HIF-activating effect (Figure 1B lower panel). Furthermore, knockdown of HIF1α expression by siRNA reduced HIF1α mRNA (Figure 1C insert) as well as the baicalein-increased Epo and VEGF expression (Figure 1C). The production of Epo and VEGF protein were also increased by baicalein treatment (Figure 1D), with the data showing that VEGF was more abundant than Epo in terms of its basal and baicalein-increased levels. Thus, neuronal Epo and VEGF production are both inducible by baicalein treatment via activation of HIF1α.

#### Involvement of 12/15-LOX in the Epo/VEGF-inducing effect of baicalein

**Figure 2:**
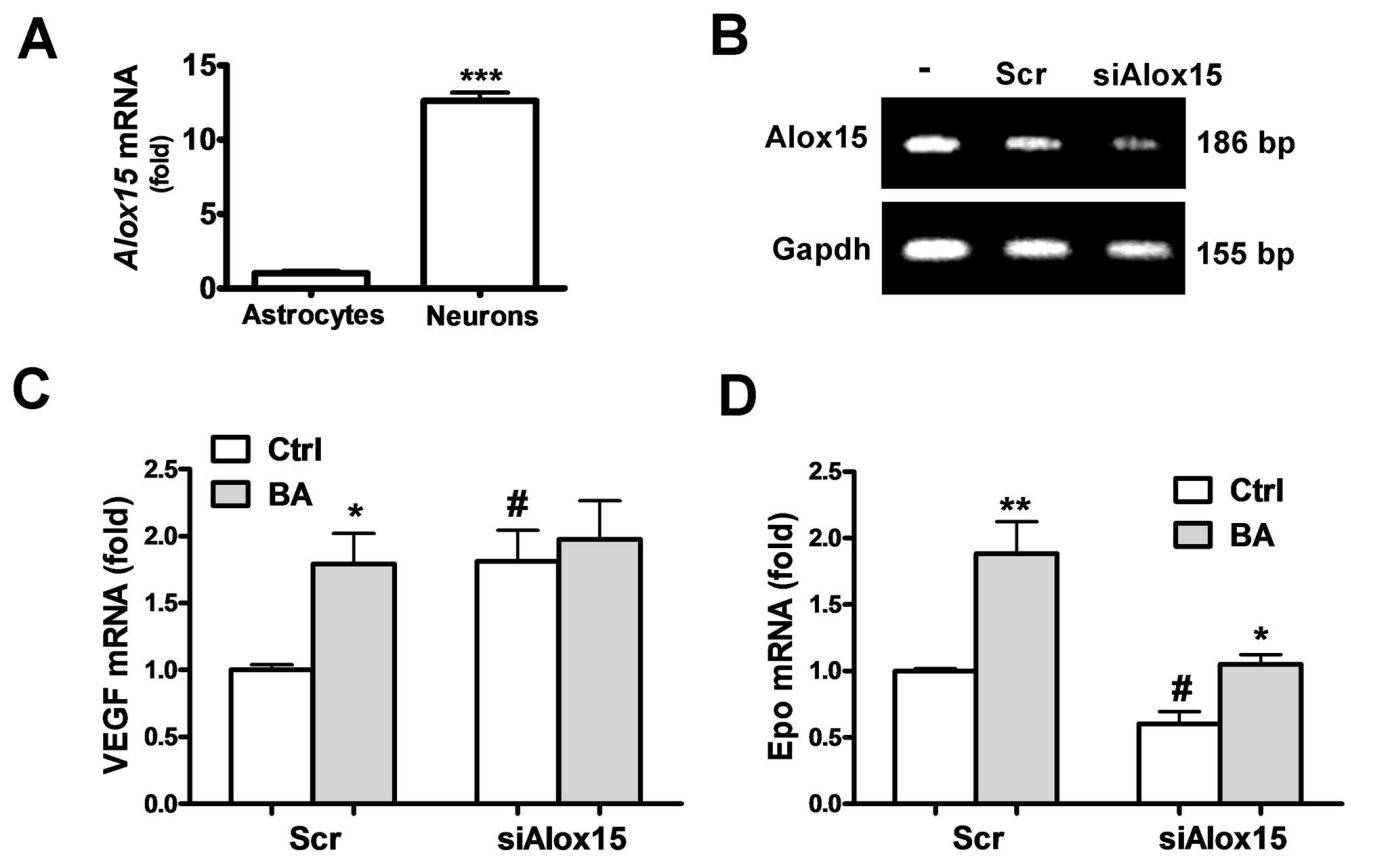
Effects of 12/15-LOX knockdown on the baicalein-induced Epo and VEGF gene expression. (A) qRT-PCR of 12/15-LOX mRNA in neurons and astrocytes. (B) RT-PCR analysis of 12/15-LOX mRNA in neurons transfected with scrambled RNA (Scr) or si*Alox15* for 72 h. (C, D) qRT-PCR of VEGF (C) and Epo (D) mRNA in siAlox15 or Scr-transfected neurons with or without 3.5 μM BA treatment for 12 h. In (A), ***p<0.01 versus Astrocytes group (n=3). In (C) and (D), *p<0.05 and **p<0.01 versus control (Ctrl); ^#^ *p*<0.05 versus the Scr-Ctrl (n=3).

Inhibition of 12/15-LOX was considered to be the major pharmacological effect of baicalein for neuroprotection. We found that primary cultured neurons had much higher 12/15-LOX expression than cultured astrocytes (Fig. 2A). Therefore, we investigated whether reduction of 12/15-LOX can simulate the Epo/VEGF-inducing effect of baicalein in neurons. Using siRNA specific for the rat *Alox15* gene (si*Alox15*) that knockdown 12/15-LOX expression (Figure 2B) to mimic the baicalein inhibition of this enzyme, we found that the VEGF expression became 1.81 fold higher than with the scrambled siRNA-transfected control. Baicalein induced a 1.79 fold increase of VEGF in the scrambled siRNA-transfected neurons, similar to the si*Alox15*-induced effect (Figure 2C). In contrast, si*Alox15* decreased Epo mRNA expression to 0.6 fold (Figure 2D). Furthermore, reduction of 12/15-LOX expression abolished the VEGF-inducing effect of baicalein, but its Epo-inducing effect was preserved at apprx.1.79 fold as compared with the induction in the scrambled control (1.9 fold). Thus, baicalein inhibition of 12/15-LOX seems to contribute to its induction of VEGF, but not Epo.

### Baicalein activates Epo and VEGF gene transcription via class I PI3K/Akt/HIF-1α signaling pathway in cortical neurons

**Figure 3:**
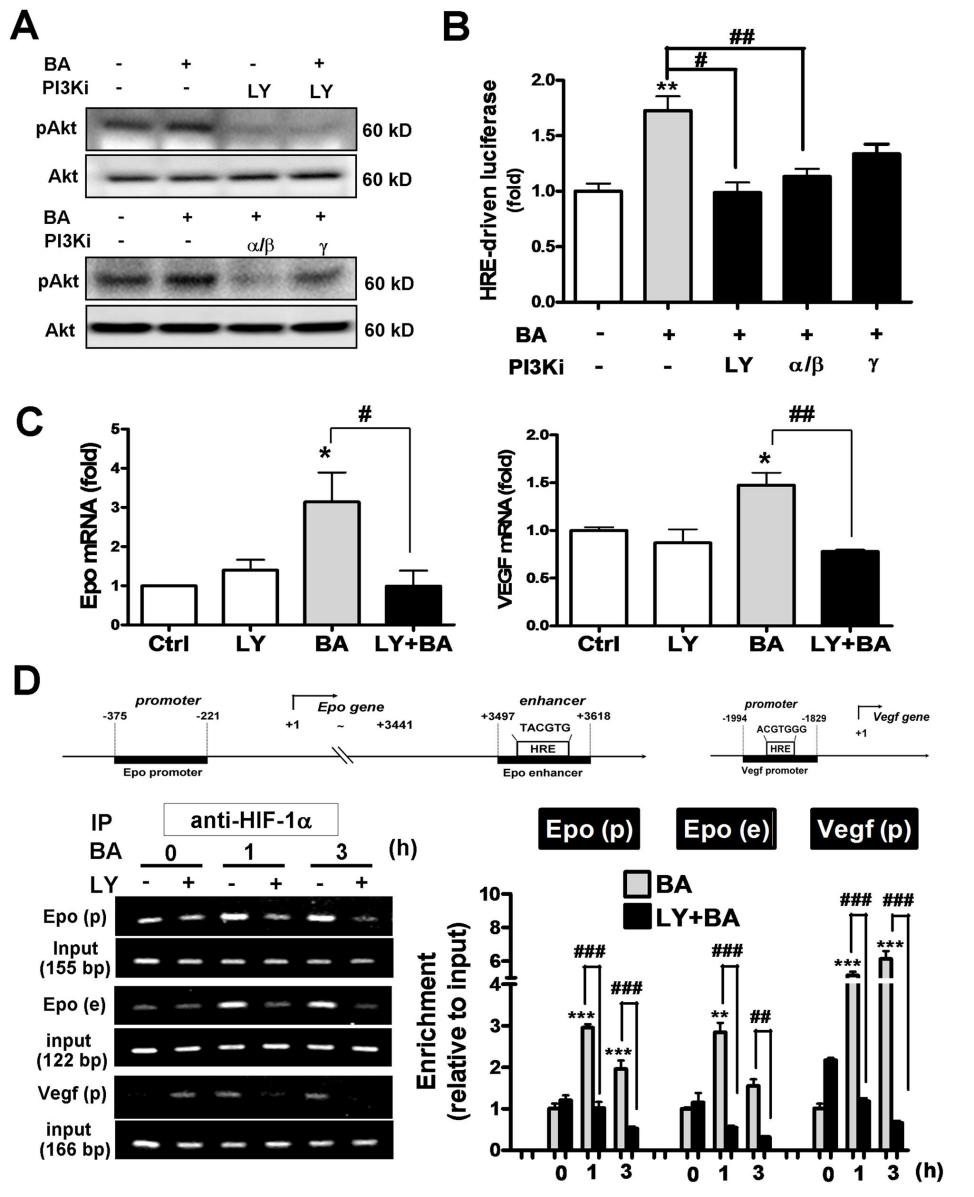
Effects of PI3K inhibitors on the baicalein–activated HIF1α and Epo/VEGF gene transcription in neurons. Neurons were treated with 3.5 μM BA with or without 1 h pretreatment with the pan PI3K inhibitor LY294002 (LY, 10 μM), PI3Kα/β inhibitor (PI3K α inhibitor-2, 50 nM), or PI3Kγ inhibitor (CAY10505, 200 nM). (A) Cells were harvested at 30 min after the BA treatment for Western blotting of pAkt and Akt. (B) Dual-luciferase activity assay of pHRE_*EPO*_-Luc expression in cells 24 h after the treatments. (C) qRT-PCR of Epo and VEGF mRNA expression in cells 24 h after the treatments. (D) Anti-HIF1α-based ChIP assay was performed at 0, 1, 3 h after the BA treatment to analyze the HIF1α-associated rat *Epo* promoter fragment (*Epo-p*), HRE-containing *Epo* enhancer fragment (*Epo-e*) and HRE-containing *Vegf* promoter fragment (*Vegf-p*) by PCR (left panel) and qPCR (right panel). *p<0.05, **p<0.01, ***p<0.001 versus control; ^#^ p<0.05, ^# #^ p<0.01, ^# # #^ p<0.001 versus the BA-treated group (n=3).

Baicalein was reported to activate Akt signaling pathway in both cultured cortical neurons and cerebral ischemia [2]. We observed similar effect that 3.5 μM baicalein increased Akt phosphorylation (Figure 3A). This effect was blocked not only by a pan PI3K inhibitor LY294002 at 10 μM, but also by a selective class IA PI3K α/β isoform inhibitor PI3K α inhibitor-2 at 50 nM (IC50=2 nM for PI3Kα, 16 nM for PI3Kβ). A class IB PI3K γ isoform inhibitor CAY10505 at 200 nM (IC50=30 nM) partially reduced the baicalein-induced Akt phosphorylation (Figure 3A). Furthermore, baicalein-induced HRE-driven reporter expression was also blocked by LY294002 and PI3K α inhibitor-2 significantly, and was less sensitive to the PI3Kγ inhibitor (Figure 3B). Baicalein-induced Epo and VEGF gene expression as well as the HIF1α binding to their gene enhancer/promoter regions were both blocked by LY294002 as revealed by the qRT-PCR and ChIP assay, respectively (Figure 3C and 3D). Notably, the three PCR-amplified HIF1α binding fragments in the *Epo* and *Vegf* genes by ChIP assay are: (1) HRE-containing regions in the *Vegf* promoter, (2) HRE-containing region in the *Epo* 3’ enhancer, and (3) CBP/p300 binding site of the *Epo* promoter that recruits HIF1α-bound enhancer (Figure 3D upper panel for gene map). Together, the data suggest that baicalein activates class I PI3K/Akt signaling, which mediates the activation of HIF1α and subsequent transcriptional activation of Epo and VEGF gene expression.

### Both extracellular Epo/VEGF neutralization and PI3K inhibitor treatment reverse baicalein neuroprotection against excitotoxicity

**Figure 4:**
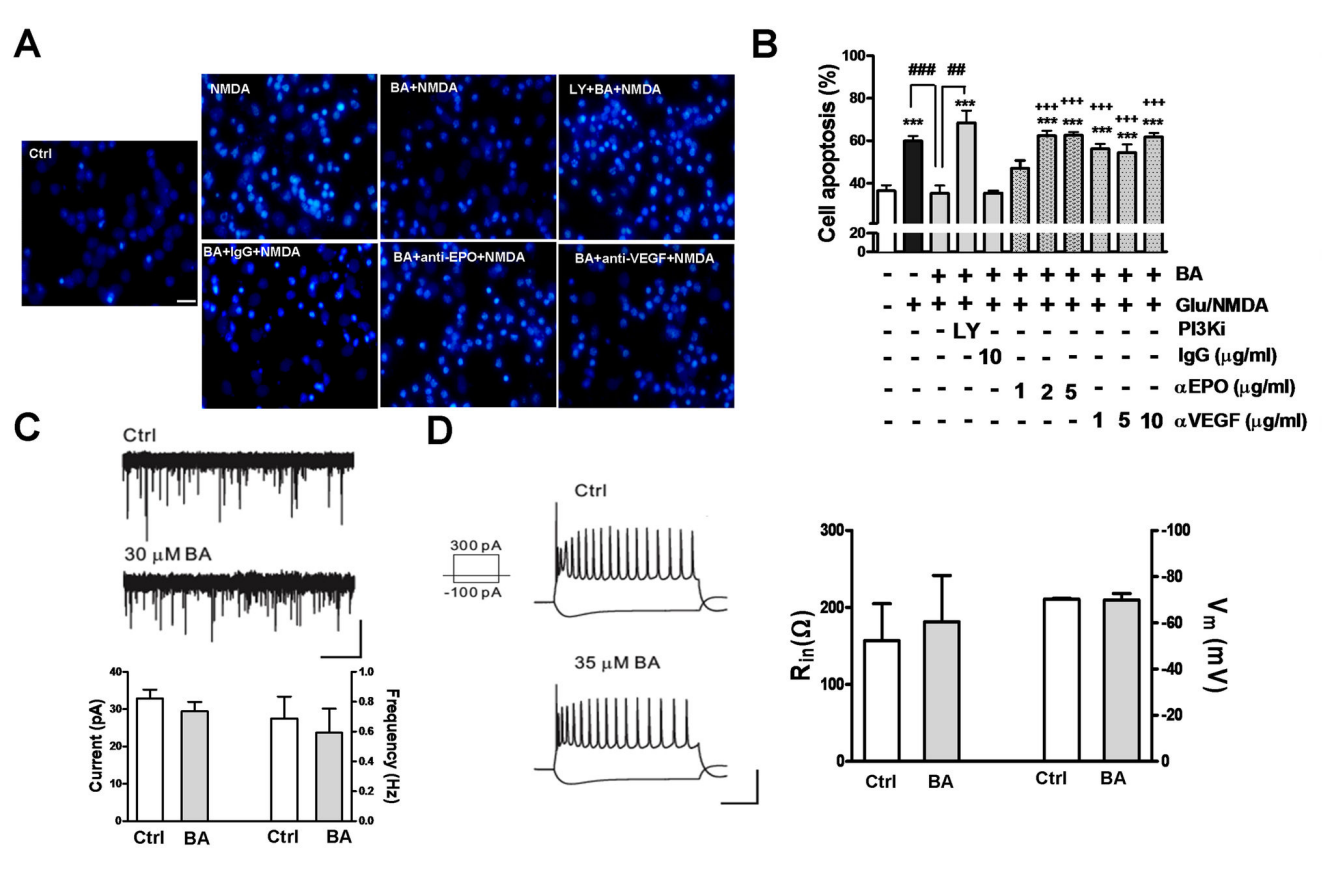
Effects of extracellular Epo/VEGF neutralization and PI3K inhibitor treatment on the baicalein neuroprotection against excitotoxicity, and baicalein effect on neuronal excitability. (A, B) Neurons were pre-treated with baicalein (BA, 3.5 μM) with or without LY294002 (LY, 10 μM), anti-EPO, or anti-VEGF, or normal goat IgG antibodies at the concentrations as indicated for 12 h, followed by the glutamate (25 μM)/ NMDA (25 μM) (Glu/NMDA) treatment. Neurons were stained with DAPI to visualize nuclear condensation for cell apoptosis. (A) Representative fluorescent micrographs with anti-Epo and anti-VEGF antibodies at 5 μg/ml; (B) quantitative result of the percent of apoptotic cells. Scale bar: 20 μm. ***p<0.001 versus vehicle control; ^# #^ p<0.01 and ^# # #^ p<0.001 versus the BA + Glu/NMDA-treated group; ^+ + +^ p<0.001 versus the BA/IgG + Glu/NMDA-treated group (unpaired *t*-test). (n=5). (C) Miniature GABA_A_-receptor-mediated currents with the example traces showing before and after bath application of BA (30 μM) (*upper* panel) and summary of BA effect on the amplitude and frequency (*lower* panel). Scale bars: 1 min/50 pA. (D) Left panel: Representative traces of membrane responses of CA1 pyramidal neurons evoked by the 1-s depolarizing (300 pA) and hyperpolarizing (-100 pA) current pulses before (Ctrl) and after BA application. Scale bars: 250 ms/50 mV. Right panel: Summary of the BA effect on membrane potential (Vm, n=3) and input resistance (Rin, n=4) of CA1 pyramidal neurons.

Glutamate excitotoxicity is the major cause of neurodegeneration. Excitotoxic 25 μM glutamate (Glu)/ 25 μM NMDA treatment that profoundly increased neuronal apoptosis, as indicated by the fluorescent imaging and quantitative data of nuclear condensation, was markedly attenuated by the 3.5 μM baicalein treatment (Figure 4A and 4B). This effect was blocked by the PI3K inhibitor LY294002, reproduced the reported mechanism of baicalein neuroprotection. We further examined whether this neuroprotective effect is attributed to its induction of Epo and VEGF production. Anti-Epo or anti-VEGF antibodies at concentrations from 1 to 10 μg/ml were applied to the media of cultured neurons followed by the baicalein pretreatment and excitotoxic Glu/NMDA stimulation to examine excitotoxicity. Data show that both antibodies, at the concentrations from 1 to 10 μg/ml, were effective in reversing the neuroprotective effect of baicalein against excitotoxicity, whereas the normal IgG at 10 μg/ml had no effect. Therefore, baicalein-induced Epo and VEGF production from neurons plays a causal role in its neuroprotective activity.

### Acute baicalein treatment does not affect inhibitory GABA_A_ receptor activity, excitatory glutamateric transmission, or neuronal excitability

Baicalein was shown to have benzodiazepine-like action on GABA_A_ receptors [31], which may influence neuronal activity affecting Akt phosphorylation [41]. Therefore, we examined whether baicalein neuroprotection against excitotoxicity involves its effect on GABA_A_ receptor activity and neuronal excitability. Our data indicate that bath application of baicalein (30μM) on hippocampal slices for 1h had no effect on the frequency and amplitude of miniature GABA_A_ receptor-mediated currents (Figure 4C), the membrane responses evoked by either depolarizing or hyperpolarizing current pulses as recorded from CA1 pyramidal cells or dentate granule cells. [Figure 4C ; summary data (n=10 cells) were pooled from CA1 pyramidal cells and dentate granule cells], the membrane responses evoked by either depolarizing or hyperpolarizing current pulses as recorded from CA1 pyramidal neurons (Figure 4D, left panel trace result). Furthermore, we examined the effect of BA on the excitatory glutamatergic transmission, and found that BA (30 μM) had no effect on the slope of fEPSP evoked at CA3-CA1 synapses (see Figure S2 in Information S1). This result is in agreement with a study by Wang et al. (2011), in which BA up to 50 μM showed no effect on the excitatory glutamatergic transmission. Analysis of the membrane potential and input resistance also shows that none of these measures were affected by the baicalein treatment (Figure 4D, right panel).

Taken together, baicalein treatment on neurons, without affecting neuronal excitability and the balance of excitation/inhibition (E/I) transmission as indicated by the lack of effect on inhibitory GABA_A_R receptor activity and excitatory glutamateric transmission, provides neuroprotection against excitotoxicity via PI3K signaling and induction of Epo and VEGF production.

### Baicalein induces Epo and VEGF expression in astrocytes

**Figure 5:**
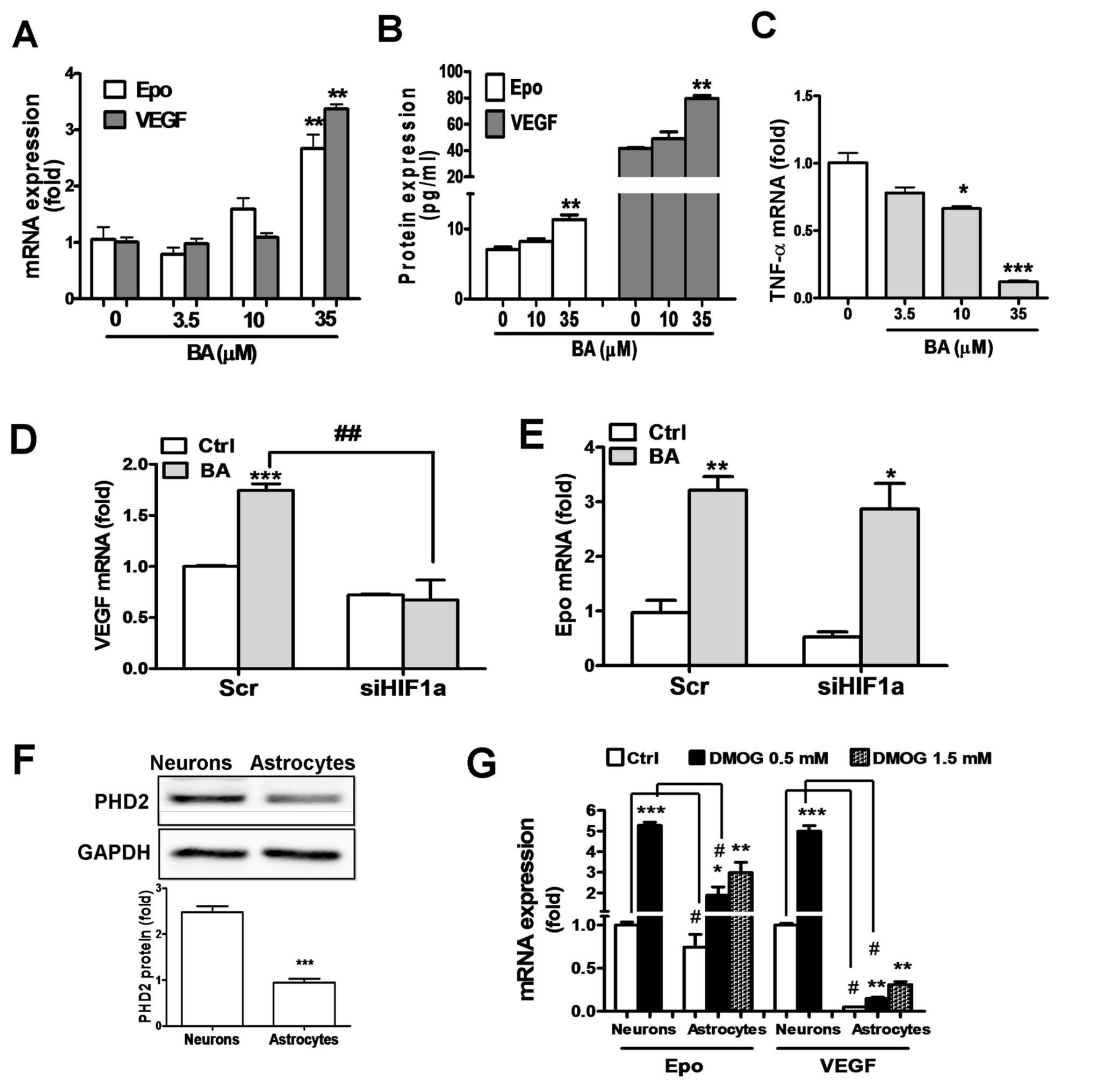
Baicalein effect on astrocytic Epo/VEGF expression and its HIF1α dependency and correlation with the PHD inhibitor effect in astrocytes. (A, C) Primary cultured astrocytes were treated with baicalein (BA) at the indicated concentrations for 24 h, followed by mRNA extraction for qRT-PCR analysis of Epo, VEGF,(A) and TNFα (C) transcripts. (B) Culture medium of astrocytes was collected 24 h after the BA treatment for ELISA analysis of Epo and VEGF. (D, E) For the HIF1α dependency experiment, the scrambled RNA- or si*Hif1a*-transfected astrocytes were treated with BA (35 μM) for 24 h, followed by qRT-PCR analysis of VEGF (D) and Epo (E) mRNA. (F) Protein levels of PHD2 in neurons versus astrocytes as analyzed by Western blotting. (G) qRT-PCR of Epo and VEGF mRNA in 0.5 mM or 1.5 mM DMOG-treated neurons and astrocytes *p<0.05, **p<0.01, ***p<0.001 versus control; ^#^ p<0.05 and ^# #^ p<0.01 versus the Scr-BA-treated group in (D), and versus Neurons-Ctrl or Neurons-DMOG group in **G** (n=3).

We further examined whether baicalein can also increase Epo and VEGF expression in astrocytes. When attempting to treat cultured astrocytes with baicalein at low micromolar concentrations effective for neurons, i.e. 3.5 and 10 μM, we found that neither mRNA nor protein levels of Epo and VEGF was increased unless the concentration was raised to 35 μM (Figure 5A and 5B). This high concentration of baicalein does not appear to stimulate proinflammatory response in astrocytes, as indicated by its dose dependent decrease rather than increase of the proinflammatory TNFα expression (Figure 5C).

Next, we examined whether baicalein-induced Epo and VEGF expression in astrocytes is also mediated by HIF1α. The data show that HIF1α knockdown abolished baicalein-induced VEGF (Figure 5D), but had no effect on the Epo induction (Figure 5E). Thus, higher concentration of baicalein is required for inducing Epo/VEGF expression in astrocytes than in neurons, and HIF1α in astrocytes only mediates the baicalein-induced VEGF, but not Epo.

### Differential PHD2 abundance and PHD inhibitor-induced Epo/VEGF expression between neurons and astrocytes

The relatively low potency of baicalein in astrocytes as reflected by the higher effective concentration could be due to the low abundance of its binding targets, such as 12/15-LOX. However, 12/15-LOX activity only involves in the baicalein-induced VEGF, but not Epo (Figure 2C and 2D). We examined whether PHD2, another baicalein binding target, is also differentially expressed in neurons versus astrocytes. Western blotting results show that PHD2 in neurons is about 2.5 fold higher than astrocytes (Figure 5F). This difference may lead to the low sensitivity of astrocytes to PHD inhibitor treatment. We tested this assumption by using a non-selective PHD inhibitor DMOG to treat neurons and astrocytes at the same concentration of 0.5 mM. The results show that 0.5 mM DMOG in neurons profoundly increased both Epo and VEGF mRNA by 5.3 folds and 5.0 folds, respectively (Figure 5G, left panel). In astrocytes, DMOG at 0.5 mM only increased Epo mRNA by 2.5 fold and VEGF mRNA by 3 fold, and the induction can be respectively raised to 4.0 and 6.2 fold when DMOG concentration was increased to 1.5 mM. Notably, both gene transcripts were increased to a much lesser degree than was found in neurons.

Since both baicalein and DMOG are more effective in inducing Epo and VEGF expression in neurons than in astrocytes, it is likely that their common mechanism of action, i.e. inhibition of PHD2, contributes at least in part to this cell type-dependent sensitivity due to the differential abundance of PHD2.

### Baicalein-treated astrocytes show neuroprotection via PI3K, but PI3K/Akt signaling does not mediate the induction of astrocytic Epo and VEGF

**Figure 6:**
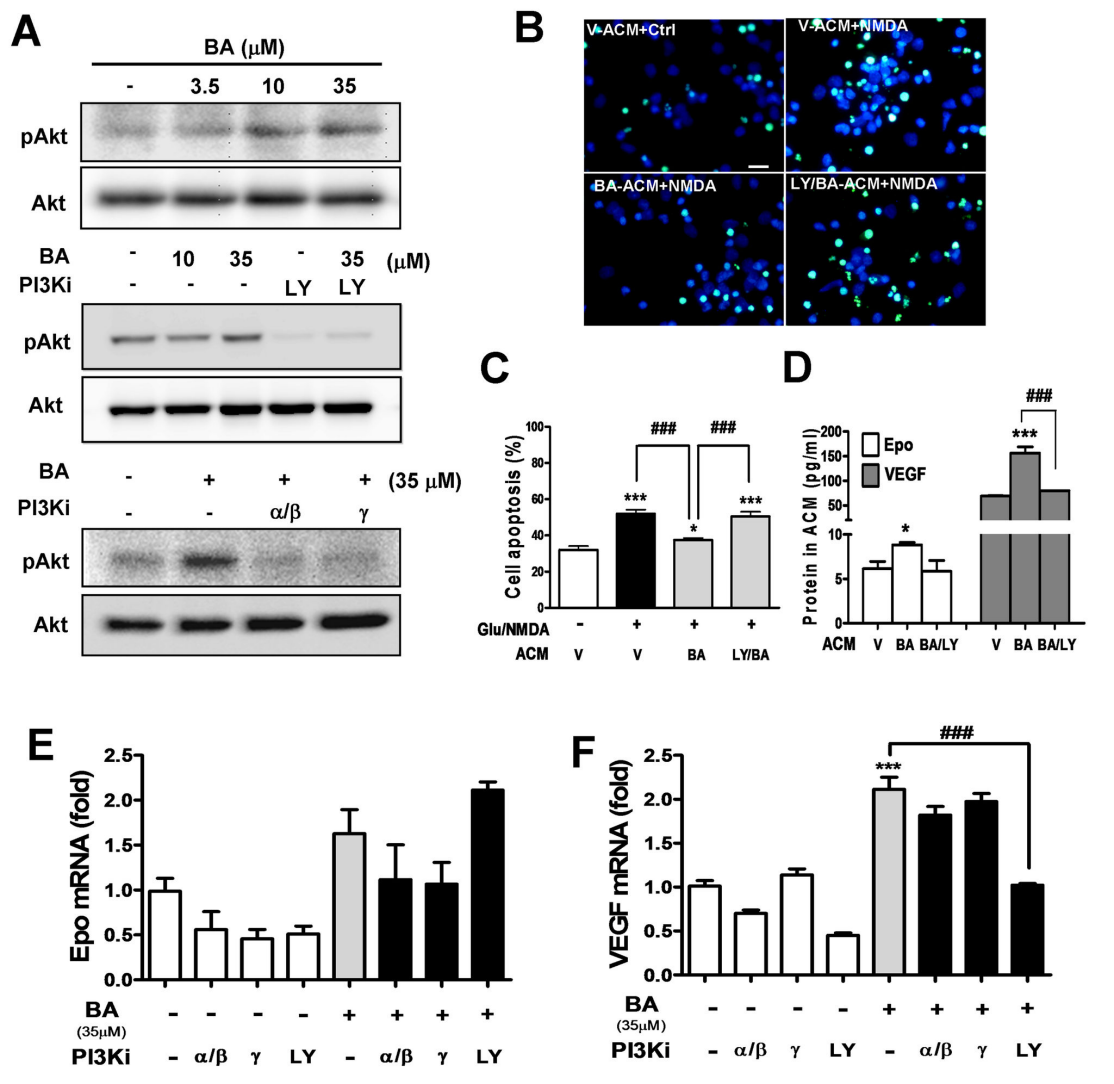
Effects of PI3K inhibitors on the baicalein-induced Akt phosphorylation, astrocyte-mediated neuroprotection, and astrocytic Epo/VEGF expression. (A, E, F) Astrocytes were treated with baicalein (BA) at indicated concentrations (3.5, 10 or 35 μM) or pretreated with LY294002 (LY, 10 μM), PI3K α β inhibitor (PI3K α inhibitor-2, 50 nM), or PI3Kγ inhibitor (CAY10505, 200 nM) for 1 h, followed by the BA treatment. (A) Total proteins were harvested 30 min after the treatment for Western blotting of pAkt and Akt. (B, C) Cortical neurons were incubated with astrocyte-conditioned medium (ACM) from astrocytes treated with 35 μM BA in the presence or absence of LY pretreatment. Three hours after the ACM incubation, neurons were treated with glutamate (25 μM)/NMDA (25 μM) (Glu/NMDA) treatment for 21 h, followed by TUNEL assay to visualize (B) and quantify (C) apoptotic cells. Scale bar in (B): 20 μm. (D) ELISA analysis of Epo and VEGF concentrations in ACM used in (B) and (C). (E, F) qRT-PCR of Epo (E) and VEGF (F) transcripts in astrocytes 24 h after the treatment. *p<0.05 and ***p<0.001 versus the respective control group; ^# # #^ p<0.001 versus the BA- or BA-ACM-treated group (n=3)..

Since the neuronal Epo/VEGF-inducing effect of baicalein for neuroprotection is mediated by PI3K signaling (Figure 3), a similar mechanism might apply to astrocytes. We examined the PI3K-mediated Akt phosphorylation in astrocytes upon baicalein treatment, and found that, similar to its effective concentration for Epo/VEGF induction, baicalein at 35 μM, but not 10 μM, was effective in inducing Akt phosphorylation (Figure 6A, top and middle panel). This effect was completely blocked by the pan PI3K inhibitor and the two class I PI3K inhibitors (Figure 6A, middle and bottom panels). To examine how baicalein-treated astrocytes affect neuronal survival and the involvement of PI3K-activating effect of baicalein, we prepared and applied baicalein-treated astrocyte-conditioned medium (ACM), with baicalein and other small molecule compounds removed (see Methods section), for incubation with cortical neurons subjected to excitotoxic glutamate/NMDA stimulation. TUNEL images (Figure 6B) and quantitative data (Figure 6C) indicated that the apoptosis rate in NMDA-stimulated neurons (52%) was significantly higher than the unstimulated neurons (32%) when both groups were incubated with vehicle-treated astrocyte-conditioned medium (V-ACM). Incubation with baicalein-ACM significantly reduced the NMDA-induced neuronal apoptosis to 37%, whereas incubation of ACM derived from baicalein-treated astrocytes pretreated with LY294002 (LY/BA-ACM) showed no significant reduction of NMDA-induced neuronal apoptosis (50%). We further confirmed the Epo and VEGF concentrations in the ACM derived from each condition as shown in Figure 6D, in which both cytokine levels in LY/BA-ACM were indeed lower than in BA-ACM, and were similar to the level in V-ACM.

Finally, we examined whether baicalein-induced astrocytic Epo/VEGF expression is also PI3K-dependent. Surprisingly, the Epo/VEGF-inducing effect of baicalein was only attenuated by the pan PI3K inhibitor LY294002 but not the selective inhibitors for class I PI3K α/β and γ isoforms (Figure 6E and 6F) although all these inhibitors abolished baicalein-induced Akt phosphorylation (Figure 6A). These results suggested that although baicalein can provide neuroprotection by inducing neurotrophic astrocytes in a LY294002-reversible manner, its Epo/VEGF-inducing effect seems to be independent of its activation of the class I PI3K-mediated Akt signaling pathway in astrocytes.

## Discussion

**Figure 7:**
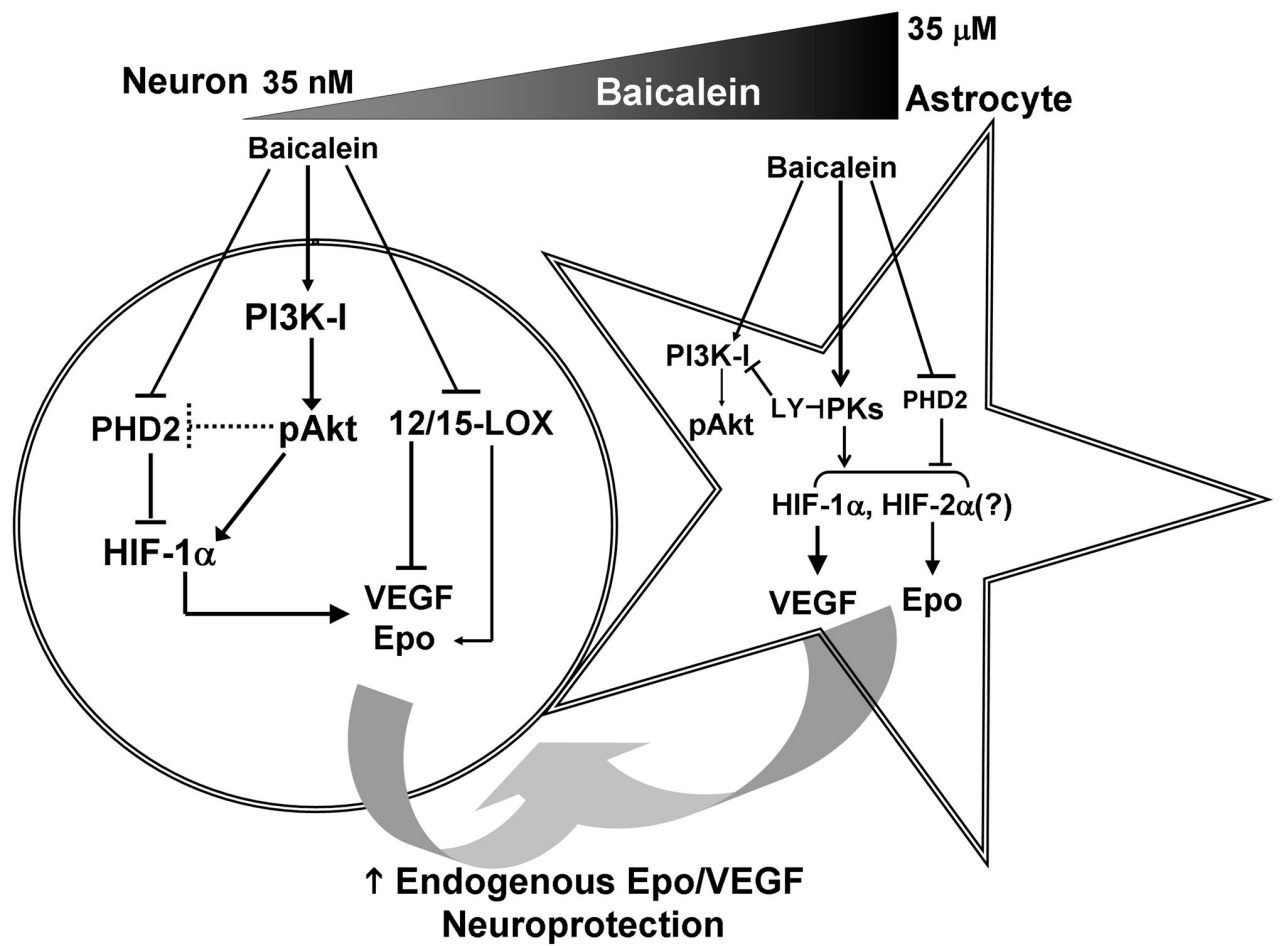
Cell type-specific signaling mechanism of baicalein-induced endogenous Epo and VEGF production from neurons and astrocytes for neuroprotection. Baicalein treatment in neurons activates class I PI3K/Akt to induce HIF1α-mediated Epo/VEGF expression with a minimal effective concentration at 35 nM. Its direct inhibition of 12/15-LOX additionally contributes to the induction of neuronal VEGF, but not Epo. In astrocytes where PHD2 is in low abundance and 12/15-LOX is lacking, high concentration of baicalein (35 μM in minimum) is required to activate class I PI3K/Akt. However, baicalein-induced upregulation of astrocytic Epo/VEGF is sensitive to LY294002 (LY) but not the selective class I PI3K inhibitors, suggesting that other LY294002-inhibitable protein kinases (PKs) mediate the effect. Furthermore, baicalein-induced astrocytic Epo expression is HIF1α-independent and possibly by HIF2α that reportedly mediates astrocytic Epo gene transcription and can also be stabilized when PHD2 is inhibited. Since brain neurons and astrocytes may access nanomolar and micromolar concentration of baicalein respectively when the compound is applied peripherally, the increased production of Epo and VEGF from both cell types may thus be converged to provide neuroprotection against excitotoxic or other neurotoxic insults.

The present study demonstrated an intriguing feature of baicalein neuroprotection via induction of Epo/VEGF production from both neurons and astrocytes with cell type-specific signaling mechanisms. First, the PI3K/Akt signaling pathway, which is mainly mediated by the class I PI3K, only contributes to the Epo/VEGF-inducing effect of baicalein in neurons, but not astrocytes. Second, baicalein activates HIF1α in a PI3K-dependent manner, and this only contributes to its Epo-inducing effect in neurons, but not in astrocytes, whereas it is required for the VEGF induction in both cell types. Third, not only 12/15-LOX but also PHD2 are much more enriched in neurons than in astrocytes, and this differential abundance seems to contribute to the cell type-specific effects of baicalein in inducing Epo and VEGF expression. Finally, this is the first report to show that baicalein-treated astrocytes can provide neuroprotection against excitotoxicity. The deduced mechanism is illustrated in Figure 7 and discussed as follows.

### PI3K dependency of baicalein –induced Epo/VEGF expression

Baicalein activates HIF1α to mediate Epo/VEGF gene expression suggesting its potential in inducing preconditioning-like effects under normoxia. Although the HIF-activating effect of baicalein could be attributed to its direct inhibition of PHD2 [9], we found that in neurons the class I PI3K/Akt signaling seems to dominate this effect and the subsequent Epo/VEGF induction. A recent report shows that PI3K/Akt pathway mediates HIF1α activation via mTOR-mediated inhibition of PHD2 in melanoma cells [14], indicating that baicalein-activated PI3K/Akt may also inhibit PHD2 in neurons. In contrast, in astrocytes the baicalein-activated Akt via class I PI3K does not contribute to its Epo/VEGF-inducing effect while the effect was inhibitable by the pan PI3K inhibitor LY294002. One possibility for this surprising finding is that the effect might be mediated by other classes of PI3Ks, i.e. class II and class III PI3Ks that do not mediate Akt phosphorylation primarily [12]. Nonetheless, evidence is still lacking regarding the relationship between the non-class I PI3Ks and HIFs. Notably, LY294002 was reported to inhibit protein kinases other than PI3Ks, such as casein kinase 2 (CK2) [42]. However, other reports showed contradictory results indicating that CK2 activity was not affected by LY294002 [43]. The identity of LY294002-sensitive protein kinases for the Akt-independent induction of astrocytic Epo/VEGF and possibly other astrocyte-derived mediators for neuronal survival requires further investigation.

### Cell type-specific dependency on HIF1α for the baicalein-induced Epo expression

HIF1α is critical for both Epo and VEGF induction in neurons, whereas in astrocytes it only involves in the VEGF-inducing effect of baicalein. It was reported that the major HIF in astrocytes that mediates hypoxia-induced Epo is HIF2α, but not HIF1α [44]. In a retinal ischemia study, HIF1α stabilization upon hypoxia was found in neuronal cells of the inner retinal layers whereas HIF2α was restricted to Müller glia and astrocytes [45]. Although both neuroprotective cytokines can be induced by baicalein in these two cell types, their differential HIF dependency in astrocytes is worth noting for the future evaluation of PHD- or HIF-based neuroprotection.

### Baicalein activates PI3K in neurons and astrocytes - possible binding targets

Although our finding on the baicalein activation of PI3K correlates well with a previous report on the baicalein-induced Akt phosphorylation in neurons and brain [2], it was also reported to inhibit PI3K/Akt signaling in cancer cells [36] and immune cells [35]. In fact, a high throughput screening for PI3K inhibitors in a cell-free system found that baicalein can inhibit PI3Kα/β by direct binding [46], which suggests that the observed baicalein activation of PI3Ks in neurons and astrocytes may not be due to their direct binding to PI3Ks. Our electrophysiology study that examined whether baicalein binding to GABA_A_ receptors may contribute to its activation of PI3K also shows negative results (Figure 4C and 4D), which contrasts with the view that baicalein could interact with the benzodiazepine binding site of GABA_A_ receptors. Other possibilities include receptor tyrosine kinases and G-protein coupled receptors that have been suggested as the primary targets of natural flavones to induce PI3K/Akt signaling [47]. Since the PI3K-activating effect of baicalein in both neurons and astrocytes plays critical role in its neuroprotective activity, the present study suggests an importance direction to identify baicalein binding targets that mediate PI3K signaling pathway in these two cell types to delineate its cell type-specific mechanism of action.

### Low abundance of 12/15-LOX and PHD2 in astrocytes: prevention of excessive astrocytic VEGF induction by baicalein

One of the important observations in this study is that both 12/15-LOX and PHD2 are expressed in much lower levels in astrocytes than in neurons. We found that baicalein inhibition of 12/15-LOX contributes to its induction of neuronal VEGF, which coincides with previous studies showing that LOX activity inhibits VEGF gene expression in skeletal muscles and prostate cancer cells [48,49]. Notably, the lack of 12/15-LOX in astrocytes might prevent baicalein from inducing excessive astrocytic VEGF production to cause BBB disruption [30]. In fact, baicalein was found protecting BBB integrity after stroke via inhibition of 12/15-LOX in cerebrovascular endothelial cells [8]. In addition, PHD2 is also expressed much less in astrocytes than in neurons, which could be beneficial because it sets a higher threshold for its inhibitors, such as baicalein and DMOG, to prevent their excessive induction of astrocytic VEGF proven to aggrevate BBB leakage in brain injury.

### Differential sensitivity between neurons and astrocytes – implication for the neuroprotective dosage of baicalein in vivo

From the previous pharmacokinetic studies, peripheral administration of baicalein at 30-60 mg/kg, which was shown to be effective for improving functional recovery in various brain injury animal models [2,5], can yield concentrations in the blood and brain tissue of healthy rats at approx. 20-40 μg/ml (74-148 μM) and 10-18 ng/ml (37-67 nM), respectively [50]. Our results show that minimal concentrations required for baicalein induction of Epo/VEGF in astrocytes and neurons are 35 μM and 35 nM, respectively. Although the concentration of baicalein in the brain tissue delivered from the periphery seems only effective in inducing neuronal but not astrocytic Epo/VEGF, its concentration in the blood, which is higher than the effective concentration for astrocytes, may affect astrocytes via their perivascular endfeet near the BBB. In addition, the nanomolar concentration of baicalein detected in the brain tissue of 60 mg/kg baicalein-treated rats can also satisfy the minimal concentration needed to induce PI3K-HIF1α activity in neurons, whereas higher neuroprotective concentrations, i.e. 3.5 and 10 μM, may require peripheral application at higher dosage or when BBB permeability is increased. From the above, it is likely to be easier for perivascular astrocytes than for brain neurons to access effective concentration of baicalein to protect neurons. Baicalein’s effect on astrocytes has been overlooked in its neuroprotective effects *in vivo*. Further investigations are needed to delineate the critical role of astrocytes in the neuroprotective and therapeutic applications of baicalein.

## Conclusion

In conclusion, the present study reveals that induction of neuronal and astrocytic Epo/VEGF production for neuroprotection can be achieved by baicalein-induced PI3K signaling. The unique cell type-specific mechanism of action, especially with multi-pathway signaling and astrocyte-mediated neuronal survival, suggests that baicalein should be more favorable than single-target compounds to provide an intercellular neurotrophic network for preventing the progressive neuronal loss in brain injury and neurodegenerative diseases.

## Supporting Information

Information S1 2 supporting figures. (DOC)
