# Supplementary material for: Cell Type-Specific Dependency on the PI3K/Akt Signaling Pathway for the Endogenous Epo and VEGF Induction by Baicalein in Neurons versus Astrocytes
Source: PLoS One. 2013 Jul 19;8(7):e69019. doi: 10.1371/journal.pone.0069019 (PMC3719842; doi:10.1371/journal.pone.0069019)
Supplement: Information S1 — (DOC) [file pone.0069019.s001.doc]

**Supporting Information**

**
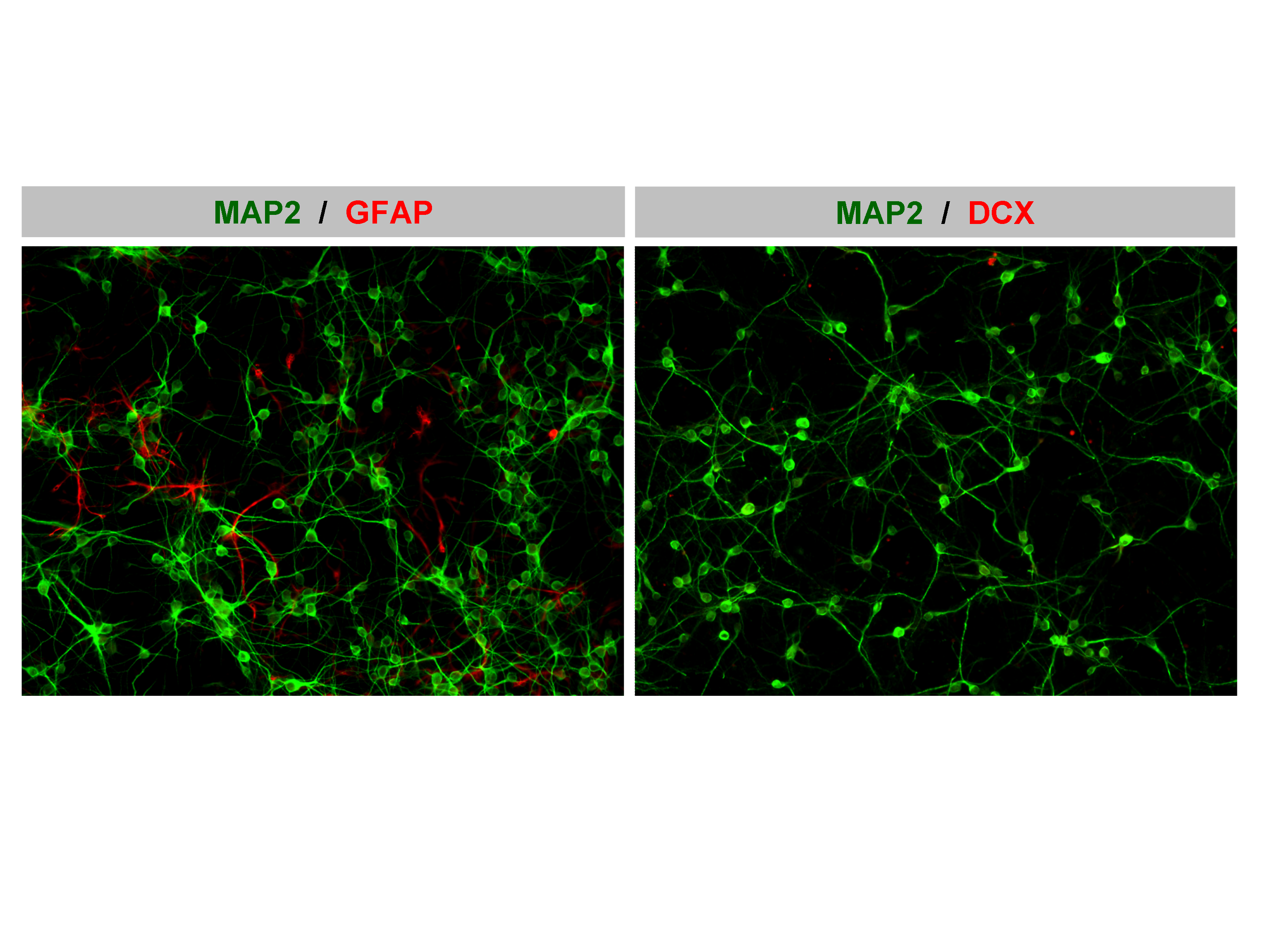
**

**Figure S1. Immunofluorescent images of cell type population in primary rat cortical neuron culture at 10 DIV.** Cultured neurons at 10 DIV were fixed and labeled with mouse anti-MAP2 (Millipore, Billerica, MA), rabbit anti-GFAP (Millipore) and guinea pig anti-doublecortin (DCX, Millipore) as indicated to respectively label neurons, astrocytes, and neural progenitor cells. Immunofluorescence development was performed using Alexa Fluor® 488-conjugated anti-mouse IgG (Invitrogen, Carlsbad, CA), Alexa Fluor® 594-conjugated anti-rabbit IgG (Invitrogen) or Alexa Fluor® 594-conjugated anti-guinea pig IgG (Jackson ImmunoResearch Laboratories, West Grove, PA). Images were taken by fluorescent microscope (Leica DM6000B), and the number of single and double-stained cells was quantified by MetaMorph 7.7 (Molecular Devices, Sunnyvale, CA). The percentages of neurons (MAP2-positive) versus astrocytes (GFAP-positive) are 86.9% and 13.1%, respectively. In MAP2-DCX double labeling, 8% of cells had detectable but weak DCX immunoreactivity, and all these cells strongly expressed MAP2.


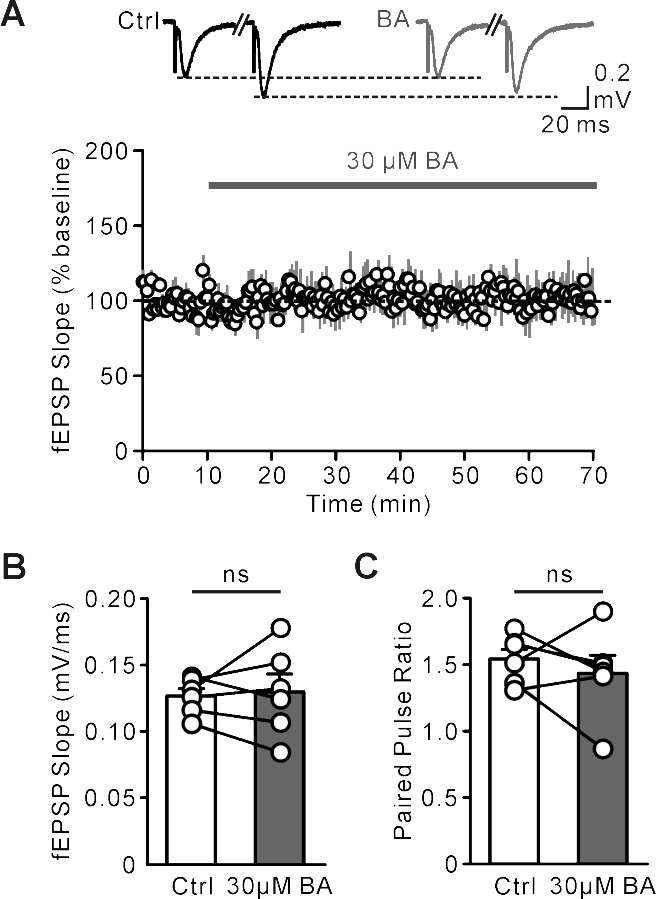


**Figure S2. Baicalein had no effect on glutamatergic transmission.**

1. Baicalein (BA, 30 μM) had no effect on basal glutamatergic transmission. After establishing a stable field excitatory postsynaptic potential (fEPSP) baseline for 10 min, BA was perfused continuously (indicated by the bar) to individual slices (n=6). Insets, representative average traces before (Ctrl) and after perfusion of baicalein (BA). Two successive fEPSPs were evoked by paired pulse stimulation with an interval of 100 ms. Average traces were obtained from the last 5-minute recordings in Ctrl and in the presence of BA, respectively.
2. Summary of fEPSP slope before and after application of BA (n=6). Controls (Ctrl), 0.13 ± 0.01 mV/ms; BA, 0.13 ± 0.01 mV/ms, n=6; *p* =1.0 by Wilcoxon signed rank test. ns, not significant.
3. Summary of paired pulse ratio of fEPSP slope before and after application of BA (n=6).
